# mHealth for chronic disease management: effects on adherence in Ghanaian patients with diabetes and hypertension

**DOI:** 10.7189/jogh.16.04203

**Published:** 2026-06-12

**Authors:** Pearl Aovare, Felix P Chilunga, Amos Laar, Nicolas Moens, Eric P Moll van Charante, Charles Agyemang

**Affiliations:** 1 Amsterdam UMC, Department of Public & Occupational Health, Amsterdam, the Netherlands; 2 University of Ghana, Department of Population, Family and Reproductive Health, Accra, Ghana; 3 Vrije Universiteit Amsterdam, Athena Institute, Amsterdam, the Netherlands; 4 Amsterdam UMC, Department of General Practice, Amsterdam, the Netherlands; 5 Johns Hopkins University, Division of Endocrinology, Diabetes, and Metabolism, Baltimore, Maryland, USA

## Abstract

**Background:**

Medication non-adherence remains a major barrier to effective chronic disease management, particularly in low- and middle-income countries. In Ghana, limited access to continuous care and patient engagement tools contributes to poor adherence among individuals living with type 2 diabetes and hypertension. Mobile health interventions, including smartphone applications, may offer scalable solutions to support long-term medication use and self-management. Here, we assess the effect of a smartphone-based intervention on medication adherence among adults with these conditions.

**Methods:**

We conducted a quasi-experimental study among 874 adults with type 2 diabetes or hypertension in Ghana. Participants were assigned to either a smartphone-based intervention, which included medication reminders, educational content, and self-monitoring tools, or standard care involving routine outpatient management and follow-up without access to the smartphone-based intervention. We measured medication adherence at baseline and six months using the Medication Adherence Rating Scale and assessed it through generalised estimating equations and difference-in-differences analyses, adjusting for baseline adherence, age, sex, and treatment setting.

**Results:**

Use of the smartphone application was associated with a greater improvement in medication adherence over six months in the overall study population (adjusted beta coefficient (a*β*) = 0.062; 95% confidence interval (CI) = 0.010–0.123). Similar improvements were observed among participants with type 2 diabetes (a*β* = 0.067; 95% CI = 0.012–0.135) and hypertension (a*β* = 0.068; 95% CI = 0.005–0.134). However, there was no statistically significant difference in adherence between the intervention and control groups at six months.

**Conclusions:**

Smartphone-based interventions were associated with modest improvements in medication adherence over time, although between-group differences at follow-up were not statistically significant. As access to mobile technology expands across Africa, such interventions may provide scalable strategies to support chronic disease management.

The burden of cardiometabolic disorders including type 2 diabetes (T2D) and hypertension is rising rapidly in low- and middle-income countries, including in sub-Saharan Africa. For instance, Global Burden of Disease studies have shown that T2D cases have increased by 56% in the last three decades from 11.3 to 17.6 per 100 000 population. The number of hypertension cases similarly increased by 30% from 19 600 to 24 500 per 100 000 population in the same period [1,2].

Despite efforts to improve the detection and treatment of cardiometabolic diseases, adherence to treatment remains suboptimal [3]. For example, adherence to diabetes treatment was reported to be 35% in Nigeria and 42% in Ghana [4], while adherence to hypertension treatment was estimated to be 38% in Kenya and 45% in South Africa [5–7] This poor adherence is a contributor to poor cardiometabolic disease control in Africa. with overall rates below 50% [8,9]. Improving adherence to prescribed medications and lifestyle recommendations, including healthy diet, physical activity, and regular follow-up care, may contribute to better glycaemic and blood pressure (BP) control.

Achieving long-term adherence to prescribed treatment regimens remains a major challenge for patients living with chronic conditions such as T2D and hypertension. Patient-related factors (*e.g.* low level of access to care knowledge, lack of motivation), medication-related factors (*e.g.* high cost, side effects), and health system factors (*e.g.* decreased low levels of access to care) can decrease levels of adherence [4,10]. The African context presents its own unique challenges in this sense; socioeconomic barriers like poverty can limit access to resources, while cultural beliefs (*e.g.* preference for herbal medication) may influence perceptions of disease and treatment effectiveness [11]. There is, therefore, an urgent need to address these underlying barriers in order to improve treatment adherence.

Internet and mobile broadband coverage continue to expand, supported by improvements in electricity access, which increased from 64% in 2010 to 86% in 2022 [12]. The widespread use of social media, with over 45% of the population actively using these platforms, further highlights the growing digital connectivity in Ghana [13–15]. Many mHealth interventions, including SMS reminders and mobile applications, do not require continuous internet connectivity, making them feasible for use in both urban and rural settings. These technologies may help address some of the ongoing challenges related to chronic disease management and medication adherence in low-resource settings. Despite the availability of treatment, disease control remains suboptimal in many African countries, with fewer than 10% of patients with diabetes and fewer than 20% of those with hypertension achieving recommended treatment targets. Medication adherence rates also remain low, averaging around 40%. These gaps highlight the potential for mHealth solutions to strengthen chronic disease management in Ghana [16,17].

Mobile health (mHealth) interventions hold considerable promise for improving cardiometabolic diseases treatment adherence. Ghana has high mobile phone penetration, with mobile subscription rates exceeding 119% of the population and approximately 55% of adults owning a smartphone. mHealth apps can enhance adherence to treatment plans by providing personalised reminders, tailored feedback, and engaging educational content, ultimately leading to better disease management [18,19]. Studies in Africa have already shown positive outcomes from mHealth interventions for cardiometabolic disease management [20,21]. For example, an SMS-based intervention in Nigeria targeting patients with T2D led to a significant 18% improvement in medication adherence over the study period [17]. Similarly, an intervention delivered in Kenya comprising a mobile app that provided medication reminders and health tips led to a 15% increase in adherence to hypertension medication [22,23]. However, these studies were of short duration, typically 12 weeks or less, and it remains unclear whether such improvements can be sustained beyond the initial engagement phase into the medium- or long-term periods required for effective cardiometabolic disease control. Furthermore, there is limited evidence from real-world studies in sub-Saharan Africa evaluating the sustained effectiveness of smartphone-based interventions over longer follow-up periods.

To address this gap, we aimed to evaluate the impact of an interactive smartphone application on self-reported medication adherence over a six-month period among adults with chronic conditions in Ghana. We hypothesised that, compared to those receiving standard care, participants using the smartphone application would demonstrate significantly higher adherence over the six-month period. We further wanted to explore whether the effect of the intervention differed between individuals with T2D and those with hypertension.

## METHODS

This is a retrospective analysis based on data from a quasi-experimental study in Ghana by our research team [24], which evaluated the impact of a smartphone-based intervention on glycaemic and blood pressure control among adults with T2D and/or hypertension (Appendix S1 in the **Online Supplementary Document**).

Here, we aimed to evaluate the effect of the smartphone-based intervention on medication adherence, a distinct behavioural and implementation outcome that was not assessed in the aforementioned quasi-experimental study. By focusing on adherence, we sought to provide mechanistic and implementation insights into how the intervention influences clinical outcomes, rather than duplicating or fragmenting the primary clinical findings. The smartphone app-based intervention included medication reminders, educational content, self-monitoring tools, and a communication platform for communication with healthcare providers.

This study was a cluster-based, non-randomised controlled trial conducted at three health facilities. Clusters were defined at the facility level to minimise contamination between intervention and control groups. Geographically separated sites were selected to minimise contamination. Two facilities were allocated to the intervention group (smartphone mHealth app) and one facility to the control group (usual care) based on geographic separation and operational feasibility. The intervention facilities were the Weija-Gbawe Municipal Hospital (urban) and the Kwahu Government Hospital (semi-rural), while the Shukura Community Hospital (urban) served as the control site. These facilities were located more than seven km apart, reducing routine interaction between patients and healthcare workers across facilities. Clinic staff were instructed not to share intervention materials across sites, and access to the mobile application was restricted to participants enrolled at the intervention facilities.

### Eligibility criteria and recruitment

We enrolled participants aged 18–85 years with confirmed T2D (HbA1c ≥6.5% or fasting glucose ≥126 mg/dL) or hypertension (BP≥140/90 mm Hg) who had received outpatient care for at least one year. We excluded individuals if they had severe comorbidities requiring recent hospitalisation; advanced cardiovascular, renal, or hepatic disease; cognitive impairment; recent significant medication changes; and if they were pregnant or breastfeeding.

Clinical records were screened to identify eligible participants between September 2022 and February 2023, who were then approached and provided written informed consent prior to enrolment. Recruitment continued until the pre-specified sample size for each facility was achieved.

### Intervention and control conditions

The intervention group received smartphones with an interactive application for T2D or hypertension management, developed collaboratively by healthcare experts, technology specialists, and end-users.

Patients were trained to log vital health metrics such as blood glucose levels, blood pressure readings, weight, and medication intake during clinic visits or home monitoring, with data securely stored and accessible to both patients and providers. Healthcare professionals used a clinician portal to review trends, communicate with patients, and adjust treatment plans in real-time. By integrating monitoring, communication, and education, the app bridged gaps between clinic visits, fostering continuous support and active patient engagement in health management. The app also included medication reminders, helping patients adhere to their prescribed treatment schedules. Participants were able to personalise these reminders according to their preferences, including timing and frequency, enhancing user engagement and adherence. Additionally, the app provided educational content covering topics such as disease management, healthy lifestyle choices, and medication usage, empowering patients with knowledge to better manage their conditions. The application supported chronic disease self-management by integrating monitoring, communication, medication reminders, and education (Appendix S1 in the **Online Supplementary Document**). Participants in the control group received standard care, which included routine clinic visits and follow-up according to facility protocols. Health measurements, including blood glucose and blood pressure readings, were recorded in paper-based logbooks during clinic visits for ongoing monitoring. The use rates of the app were carefully monitored to evaluate whether participants actively engaged with its features, such as monitoring blood glucose levels, blood pressure readings, weight, receiving medication reminders, and accessing educational content.

### Outcome measures and data collection

We assessed adherence to treatment using the Medication Adherence Rating Scale (MARS), a validated self-reported tool for evaluating adherence behaviours [25]. The MARS comprises 10 items designed to capture various dimensions of medication-taking behaviours, including intentional and unintentional non-adherence, attitudes towards medication, and forgetfulness (Appendix S2 in the **Online Supplementary Document**). Each item in the MARS is scored on a binary scale (yes = 1, no = 0). We coded items 1–6 and 9–10 such that ‘no’’ indicated adherence (1 point) and ‘yes’ indicated non-adherence (0 points). Items 7 and 8 were reverse-coded, with ‘yes’ indicating adherence (1 point) and ‘no’ indicating non-adherence (0 points). Total scores could thus range from 0 to 10, with higher scores indicating better adherence. This approach is consistent with previous studies and facilitates valid interpretation and comparison of adherence levels [26–29]. While MARS has been applied in several studies in Ghana, it has not been formally validate in Ghana.

Baseline characteristics included age (in years), sex (male or female), marital status (single, married, divorced, or widowed), education level (no formal education, primary, secondary, or tertiary), and religious affiliation (Christian, Muslim, or other. Physical activity was assessed using the Global Physical Activity Questionnaire, which classifies intensity levels as low, moderate, or high, and has been previously validated for use in Ghana [30,31]. Alcohol use was assessed based on frequency and quantity, whereby participants responded with ‘no, never’, ‘yes’, or ‘no, but used to’ regarding consumption. For current or past drinkers, participants reported the type and quantity of alcoholic beverages consumed per day, week, or month, allowing for a more detailed, quantitative assessment. Smoking status was categorised as current smoker, past smoker, or non-smoker. Additionally, clinic visits were measured by the number of visits per month. Medication use was assessed from patient medical records, including details of prescribed medications, dosages, and duration of therapy (Appendix S2 in the **Online Supplementary Document**).

### Follow up measurements (six months)

Follow-up occurred six months post-recruitment, using the same tools as at baseline (Appendix S2 in the **Online Supplementary Document**). These included medication adherence, sociodemographic factors (marital status, education, religion), lifestyle behaviours (physical activity, alcohol use, smoking), and clinical indicators such as glycated haemoglobin (HbA1c), blood glucose, blood pressure, and weight.

### Statistical analysis

#### Analytical approach

We assessed the distribution of the data prior to analysis to determine how to summarise variables and which statistical tests to select. We summarised baseline characteristics as means (x̄) and standard deviations (SDs) for continuous variables and percentages for categorical variables. We addressed missing data (<5%) by inflating the sample size by 5% at study onset. We accounted for baseline differences between groups, *e.g.* age, education, marital status, physical activity, and body mass index (BMI) in all analyses.

#### Difference-in-differences analysis

To account for repeated measurements within individuals and clustering at the facility level, we performed a secondary analysis using a difference-in-differences approach with generalised estimating equations and robust standard errors. We included a group-by-time interaction term to estimate the intervention effect, setting up three models with an exchangeable correlation structure to account for within-participant correlation and clustering by site. We adjusted for baseline imbalances between study arms in terms of age, sex, education, marital status, physical activity, BMI, and baseline adherence in multivariable models. Specifically, model 1 was unadjusted; model 2 was adjusted for age, sex, and baseline adherence; and model 3 was further adjusted for treatment facility. We present their results as beta coefficients with 95% confidence intervals (CIs) and corresponding *P*-values. We initially planned to perform subgroup analyses by sex and disease type, but did not undertake them because of insufficient statistical power.

We performed all analyses in SPSS, version 22.0 (IBM Corp., Armonk, New York, USA). A two-tailed alpha level of 0.05 was the threshold for statistical significance.

## RESULTS

### Baseline characteristics

A total of 618 participants had T2D (308 in the intervention and 310 in the control group) and 256 had hypertension (134 in the intervention and 122 in the control group). There was no overlap between type T2D and hypertension participants. Only 19 people were lost to follow up, 4/442 in the intervention and 15/432 in the control arm.

**Figure 1:**
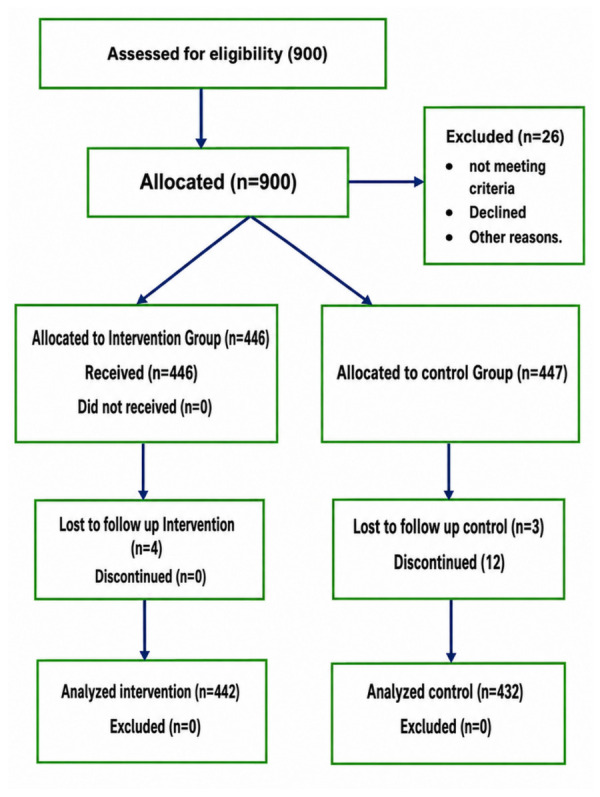
Flow diagram of participant selection, group assignment, follow-up, and analysis.

We compared baseline characteristics between 442 participants who used the mHealth app and 432 who did not (**Figure 1**, **Table 1**). The mean age differed significantly between the intervention and the control group, with the latter being older, though the overall difference was small. Educational attainment likewise differed, with a higher proportion of participants in the intervention group having no formal education (32.6%) compared to those in the control group (10.6%). Most participants in both groups were married, although this proportion was significantly higher in the intervention group (94.1% *vs*. 63.7%). Physical activity levels differed, with fewer in the intervention group reporting high activity (27.1% *vs*. 34.7%) and more reporting low activity (18.6% vs. 0.9%). The intervention group also had a significantly lower BMI (x̄ = 29.26, SD = 3.65) than the control group (x̄ = 32.16, SD = 3.82), though there was no differences in waist circumference. Clinical biomarkers (systolic BP (SBP), diastolic BP (DBP), glycated haemoglobin (HbA1c), low-density lipoprotein cholesterol (LDL-C), high-density lipoprotein cholesterol (HDL-C), and triglycerides) did not differ significantly between groups.

**Table 1:** Participants’ baseline characteristics by smartphone use

	No mHealth app use (n = 432)	mHealth app use (n = 442)	Total (n = 874)	*P*-value
**Demographics**				
Age in years, x̄ (SD)	59.0 (12.0)	58.3 (14.2)	58.6 (13.1)	0.002
Sex, n (%)				0.731
*Female*	274 (63.4)	275 (62.2)	549 (62.2)	
*Male*	158 (36.6)	167 (37.8)	325 (37.2)	
Education, n (%)				<0.001
*None*	46 (10.6)	144 (32.6)	190 (21.7)	
*Primary*	129 (29.9)	165 (37.2)	294 (33.6)	
*Secondary*	192 (44.4)	91 (20.6)	283 (32.4)	
*Tertiary*	65 (15.0)	42 (9.5)	107 (12.2)	
Marital status, n (%)				0.005
*Never married*	148 (34.3)	24 (5.4)	172 (19.7)	
*Married*	275 (63.7)	416 (94.1)	691 (79.1)	
*Ever married*	9 (2.1)	2 (0.5)	11 (1.3)	
Religion, n (%)				<0.001
*Christian*	356 (82.4)	442 (100.0)	798 (91.3)	
*Muslim*	76 (17.6)	0 (0.0)	76 (8.7)	
**Lifestyle factors**				
Smoking, n (%)				0.745
*Yes*	0(0.0)	2 (0.5)	2 (0.2)	
*No*	426 (98.6)	432 (97.7)	858 (98.2)	
*Past use*	6 (1.4)	8 (1.8)	14 (1.6)	
Alcohol intake, n (%)				0.023
*Yes*	12 (2.8)	12 (2.7)	24 (2.7)	
*No*	393 (91.0)	418 (94.6)	811 (92.8)	
*Past use*	27 (6.2)	12 (2.7)	39 (4.5)	
Physical activity, n (%)				<0.001
*High*	150 (34.7)	120 (27.1)	270 (30.9)	
*Moderate*	278 (64.4)	240 (54.3)	518 (59.3)	
*Low*	4 (0.9)	82 (18.6)	86 (9.8)	
Energy in kJ/d, x̄ (SD)	5.55(1.60)	5.90(1.55)	5.95(1.59)	<0.001
Anthropometry, x̄ (SD)				
*BMI*	32.2 (3.8)	29.3(3.7)	30.7 (3.7)	<0.001
*Waist circumference in cm*	95.88 (3.23)	96.74 (3.68)	96.35 (3.51)	0.054
*Hip circumference in cm*	99.64 (3.33)	101.83 (10.19)	100.28 (3.66)	<0.001
Clinical biomarkers (hypertension) in mmHg, x̄ (SD)				
*SBP*	152.42(9.97)	153.39(8.98)	152.93(9.46)	0.105
*DBP*	89.61(6.26)	89.43(8.78)	89.75(9.86)	0.811
Clinical biomarkers for T2D group				
*HbA1c in mmol/mol*	9.63(1.15)	9.63(1.08)	9.48(1.26)	0.998
*LDL in mmol/L*	1.517(0.41)	1.510(0.40)	1.50(0.43)	0.923
*HDL in mmol/L*	1.55 (0.26)	1.42 (0.23)	1.50 (0.25)	0.647
Triglycerides in mmol/L	2.40 (1.06)	2.36 (1.04)	2.39 (1.01)	0.820
Disease type				0.303
*Diabetes*	310 (71.8)	308 (69.7)	618 (70.7)	
*Hypertension*	122 (28.2)	134 (30.3)	256 (29.3)	

BMI – body mass index, DBP – diastolic blood pressure, HbA1c – glycated haemoglobin, HDL – high-density lipoprotein, LDL – low-density lipoprotein, SBP – systolic blood pressure, SD – standard deviation, x̄ – mean

We observed consistent app engagement in the intervention group, with total daily usage ranging from 1.0 to 2.5 hours per participant either weekly or twice weekly, with key features such as monitoring reminders and clinic appointment scheduling being actively utilised (Appendix S3 in the **Online Supplementary Document**).

### Change in adherence over six months

#### Total population

**Figure 2:**
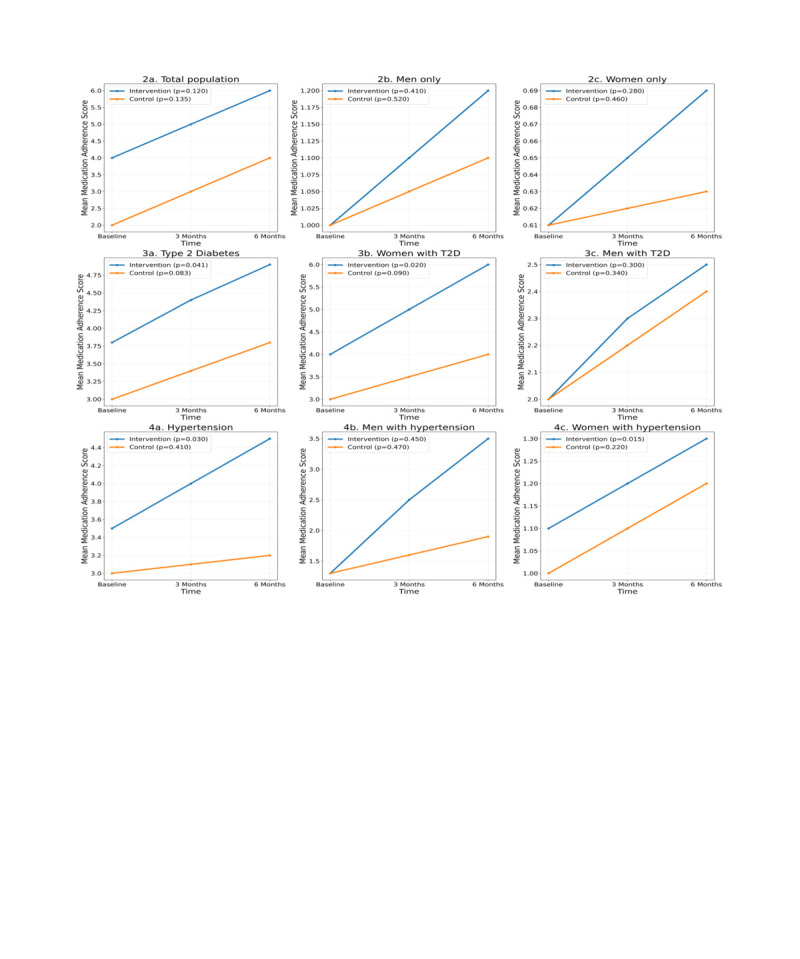
Adherence scores are presented at baseline, three months, and six months for participants using and not using a smartphone application. **Panel A.** Total population. **Panel B.** Men only. **Panel C.** Women only. **Panel D.** Total population with T2D. **Panel E.** Women with T2D. **Panel F.** Men with T2D. **Panel G.** Total population with hypertension. **Panel H.** Women with hypertension. **Panel I.** Men with hypertension. CI – confidence interval, T2D – type 2 diabetes.

Mean medication adherence in the overall population increased by 2.0 MARS points in both the intervention group (from 4.0 to 6.0) and the control group (from 2.0 to 4.0), indicating no difference in improvement between study arms. Adherence among men increased by 0.2 points in the intervention group and 0.1 points in the control group. Adherence among women improved by 0.08 points (from 0.61 to 0.69) in the intervention group, compared to 0.02 points (from 0.61 to 0.63) in the control group, suggesting a slightly greater gain with the intervention group. However, the between-group difference was not statistically significant (**Figure 2**, Panels A–C).

#### Type 2 diabetes

Adherence among participants with T2D increased by 1.1 points in the intervention group (from 3.8 to 4.9) and by 0.8 points in the control group (from 3.0 to 3.8), suggesting a modestly greater, albeit statistically non-significant improvement with the intervention. Adherence among women with T2D increased by 2.0 points in the intervention group (from 4.0 to 6.0) and by 1.0 point in the control group (from 3.0 to 4.0), indicating a greater gain among mHealth users. Adherence among men with T2D improved slightly in both groups: from 2.0 to 2.5 in the intervention group, and from 2.0 to 2.4 in the control group, showing only a marginal, statistically non-significant difference between groups (**Figure 2**, Panels D–E).

#### Hypertension

Medication adherence increased among participants with hypertension by 1.0 point in the intervention group (from 3.5 to 4.5) and by 0.2 points in the control group (from 3.0 to 3.2). The adjusted between-group difference was statistically significant. Regarding adherence among men with hypertension, the intervention group showed a notable increase of 2.2 points (from 1.3 to 3.5) compared to a 0.6-point increase in the control group (from 1.3 to 1.9), suggesting a substantially greater increase in adherence among male mHealth users. Adherence among women with hypertension increased by 0.2 points in both the intervention (1.1 to 1.3) and the control group (1.0 to 1.2), indicating no meaningful difference between groups (**Figure 2**, Panels G–I).

### Effect of smartphone use on medication adherence: difference-in-difference analysis at six-month follow-up

#### Total population

Smartphone use was associated with improved medication adherence in the total study population (adjusted difference-in-differences model; adjusted beta coefficient (a*β*) = 0.062; 95% CI = 0.010–0.123) (**Figures 2**, Panel A–C, **Table 2**).

**Table 2:** Effect of m-Health on changes in medication adherence in the total population (both type 2 diabetes and hypertension, men and women combined, n = 874) over six months (difference-in-difference estimation), a*β* (95% confidence interval)*

	Model 1	Model 2	Model 3
**No smartphone use (n = 432)**	ref	ref	ref
**Smartphone use (n = 442)**	0.072 (0.005, 0.138)	0.064(0.012, 0.125)	0.062 (0.010, 0.123)

GEE – generalised estimating equations, T2D – type 2 diabetes, ref – reference

*Model 1 was the unadjusted (crude) model. Model 2 was adjusted for baseline adherence score, age, and sex. Model 3 was adjusted for all variables included in model 2, plus treatment facility.

#### T2D

Smartphone use was associated with improved medication adherence in the total sample of participants with T2D (a*β* = 0.067; 95% CI = 0.012–0.135) (**Table 2**), as well as among men (a*β* = 0.064; 95% CI = 0.017–0.127) and women (a*β* = 0.065; 95% CI = 0.017–0.131) (**Figure 2**, Panel D–F, **Table 3**).

**Table 3:** Effect of m-Health on changes in medication adherence among adults with type 2 diabetes (n = 618) over six months (difference-in-difference estimation), a*β* (95% confidence interval)*

	Model 1	Model 2	Model 3
**No smartphone use (n = 310)**	ref	ref	ref
**Smartphone use (n = 308)**	0.070 (0.010, 0.141)	0.068 (0.014, 0.136)	0.067 (0.012, 0.135)

GEE – generalised estimating equations, T2D – type 2 diabetes, ref – reference

*Model 1 was the unadjusted (crude) model. Model 2 was adjusted for baseline adherence score, age, and sex. Model 3 was adjusted for all variables included in model 2, plus treatment facility.

#### Hypertension

Smartphone use was associated with improved medication adherence in the total sample of participants with hypertension (a*β* = 0.068; 95% CI = 0.005–0.134), as well as among men among men (a*β* = 0.056; 95% CI = 0.012–0.121) and women (a*β* = 0.034; 95% CI = 0.005–0.101) (**Figure 2**, Panel G–I, **Table 2**; Table S4 in the **Online Supplementary Document**).

### Adjusted mean MARS scores and absolute differences between smartphone users and non-users at six-month follow-up

Mean MARS scores were higher among smartphone users compared to non-users across both T2D and hypertension groups, as well as in both men and women. However, despite the significant improvements observed in the difference-in-differences analyses, the absolute between-group differences in adjusted mean MARS scores at six months were not statistically significant (Tables S5 and S6 in the **Online Supplementary Document**).

## DISCUSSION

We assessed whether a smartphone application with integrated features such as reminders, educational content, and self-monitoring tools for tracking health indicators like blood glucose and BP could improve medication adherence among adults with T2D and hypertension in Ghana over a six-month period. Medication adherence showed a modest, but statistically significant improvement in the intervention group compared with the control group. In subgroup analyses, smartphone use was associated with improved medication adherence among participants with both T2D and hypertension, suggesting that smartphone-based interventions may support chronic disease management in this setting. However, the absolute changes in adherence scores were small, and it remains uncertain whether these improvements translate into clinically meaningful clinical benefits, such as better glycaemic or BP control.

Studies have indicated that over two-thirds of app users disengage within the first 90 days of starting a new activity [32,33]. While maintaining engagement at six months is encouraging, Beishuizen and colleagues’ systematic review suggests that 12 months may represent a critical ‘tipping point’ for sustained impact of digital interventions targeting cardiovascular risk factors [22]. Therefore, while our findings suggest modest improvements in adherence over six months, we cannot determine whether these effects would be sustained beyond this period, nor whether they would translate into long-term clinical outcomes such as glycaemic or BP control in similar low- and middle-income countries. However, a recent review found that digital health strategies like reminders, feedback, goal setting, gamification, and social support can significantly improve self-care adherence in people with chronic conditions, particularly when assessed over longer periods [34].

Adherence scores among individuals with T2D improved by 60% in the intervention group and 50% in the control group (10% difference). Previous research supports the idea that individuals with T2D often exhibit higher engagement with self-management behaviours and digital tools [35,36]. However, most of these studies were of short durations (typically less than three months), limiting their ability to assess sustained engagement and medium to long-term behavioural change. For instance, Asante *et al*. [37] conducted a 12-week nurse-led mobile phone intervention in Ghana which improved glycaemic control and self-management behaviours. However, the short duration of their intervention did not allow for insights into mid-term adherence and engagement over time. In contrast, the structured reminders, real-time tracking, and personalised health recommendations offered by our intervention likely facilitated more sustained behaviour change. Conversely, a study conducted in South Africa and Malawi employing one-way SMS reminders over a similar three-month period found limited effects on glycaemic control, although modest cardiovascular benefits were reported [38]. In our study, the app’s interactive features may have contributed to stronger adherence effects outcomes by fostering user engagement, enhancing self-monitoring, and enabling timely feedback and support, and by creating a sense of accountability and personalised care. These findings highlight the importance of bidirectional communication and interactive engagement features in mHealth design. Further research on into underlying mechanisms such as patient engagement, digital self-efficacy, and health literacy could help clarify deepen our understanding of how these improvements are achieved [39].

Adherence among participants with hypertension improved significantly in the intervention group, but remained unchanged in the control group. This aligns with findings from the SMS-Text Adherence suppoRt trial in South Africa, which showed demonstrated modest reductions in systolic BP, but no significant difference between intervention and control arms [40]. Similarly, a nurse-led community-based hypertension programme in Ghana that incorporated digital support with in-person follow-ups led to improved BP control, although the intervention was not exclusively digital in design [41]. The effectiveness of the intervention may be attributed to its interactive features, structured self-monitoring, and patient-centred design, which encouraged regular engagement and supported independent self-management. The sustained effect observed at six months suggests that the app was successfully integrated into participants’ daily routines, helping to reinforce medication-taking behaviours and ongoing disease self-management.

We also noted some gender-based differences, with men showing slightly greater adherence improvements than women. This may reflect differing levels of digital literacy, healthcare-seeking behaviours, and access to mobile technologies, consistent with other prior studies in sub-Saharan Africa [42,43]. Evidence also suggests that men tend to engage more readily with technology digital tools than women, which may further could partly explain observed gender disparities in digital health intervention the differential uptake [44,45]. For instance, a study in Uganda found that an SMS-based intervention resulted in a two-fold higher response rate among men compared to women, highlighting the influence of gender disparities in access to and engagement with mHealth interventions [46]. These gender differences suggest a need for tailored, gender-sensitive mHealth strategies that address women’s digital inclusion and empowerment.

### Implications for clinical practice

Our findings suggest that smartphone-based digital interventions have the potential to support medication adherence in chronic disease management. Their integration into routine care may help promote self-management, patient engagement, and continuity of care, particularly in resource-constrained settings. Integrating such tools into routine care could complement existing health services by promoting self-management and reduce the need for frequent in-person follow-ups. Clinicians could consider incorporating mHealth applications into care plans, supported by user training and literacy initiatives to ensure accessibility and sustained engagement. However, given the exploratory nature of this study, further research with larger, randomised cluster trials is needed to confirm these benefits and guide broader implementation.

### Strengths and limitations

The quasi-experimental design of our study, with intervention and control groups, strengthens causal inference on smartphone use and medication adherence. Site-based separation minimised contamination, diverse clinic settings enhanced the relevance of the findings, and medication adherence was assessed using the validated MARS tool. The intervention incorporated multiple behaviour-support components, including reminders, educational content, communication features, and self-monitoring tools, which may have enhanced participant engagement and adherence. Methodologically, the use of generalised estimating equations enabled the analysis to account for clustering by study site and repeated measurements within participants, thereby strengthening the robustness of the findings.

Some limitations of this study should be noted. We relied on the MARS questionnaire when collecting adherence data, which is based on participants’ self-reporting their behaviour. Their responses thus may have been affected by recall and social desirability bias, particularly because participants knew they were using an adherence-supporting digital intervention. This potential bias should be considered when interpreting the reported adherence outcomes. However, we also collected and analysed objective clinical indicators, including HbA1c levels and blood pressure measurements, to strengthen the interpretation of adherence outcomes and provide indirect validation of reported adherence improvements. Nevertheless, future studies should incorporate objective adherence measures such as pharmacy refill data or electronic monitoring to enhance accuracy. Other limitations include a short six-month follow-up, potential selection bias, limited digital access, and all participants identifying as Christian, which may affect generalisability. The small number of clusters and non-random site assignment further limit causal conclusions. Although some effects on adherence may emerge by four months, the six-month follow-up is insufficient to determine whether improvements are sustained or translate into meaningful clinical outcomes. Longer-term studies of 12 months or more are needed to assess whether smartphone-based interventions lead to durable improvements in self-care and clinical outcomes in low-resource settings. Despite these limitations, our findings provide preliminary evidence to inform larger trials and future research on long-term impacts and equitable digital health engagement.

## CONCLUSIONS

This study shows that smartphone-based interventions produced small, but measurable improvements in medication adherence among adults with T2D and hypertension. Although these findings suggest that mobile technology can support chronic disease management, the modest magnitude of change raises uncertainty about whether such improvements translate into meaningful clinical benefits, including better glycaemic or blood pressure control. Future studies should assess strategies to enhance user engagement, integrate app-based tools into routine clinical care, and assess long-term clinical outcomes to maximise the potential of digital health interventions in low-resource settings.

## Additional material


Online Supplementary Document
