# Supplementary material for: mHealth for chronic disease management: effects on adherence in Ghanaian patients with diabetes and hypertension
Source: J Glob Health. 2026 Jun 12;16:04203. doi: 10.7189/jogh.16.04203 (PMC13261324; doi:10.7189/jogh.16.04203)
Supplement: Online Supplementary Document [file jogh-16-04203-s001.pdf]

Supplement to: Aovare P, Chilunga FP, Laar A, Moens N, van Charante EPM, Agyemang C. mHealth for chronic disease management: effects on adherence in Ghanaian patients with diabetes and hypertension. J Global Health. 2026;16:04203.

Table S1. Effect of smartphone use on changes in medication adherence among adults with hypertension over six months.

		**Model 1*** **β (95% CI)**	**Model 2†** **β (95% CI)**	**Model 3‡ β (95% CI)**
**Hypertension(N=256)**				
	No Smart phone use (n=122)	Ref	Ref	Ref
	Smart phone use (n=134)	0.035 (-0.042, 0.110)	0.070 (0.008, 0.135)	0.068 (0.005, 0.134)

Footnotes:

**Unadjusted (crude model).*

†Adjusted for baseline adherence score, age, and sex.

‡Adjusted for treatment facility.

Abbreviations:

CI – confidence interval, GEE – generalized estimating equations, Ref – reference category.

Table S2 Change in Medication Adherence Over Six Months

Population / Subgroup	Study Group	Baseline Adherence	Follow-up Adherence	Change (Δ)	p-value
Total Population	Intervention	4.0	6.0	+2.0	0.120
	control	2.0	4.0	+2.0	0.135
Men (Overall)	Intervention	0.70	0.90	+0.20	0.410
	control	0.70	0.80	+0.10	0.520
Women (Overall)	Intervention	0.61	0.69	+0.08	0.280
	control	0.61	0.63	+0.02	0.460
Type 2 Diabetes (T2D)	Intervention	3.8	4.9	+1.1	0.041
	control	3.0	3.8	+0.8	0.083
Women with T2D	Intervention	4.0	6.0	+2.0	0.020
	control	3.0	4.0	+1.0	0.090
Men with T2D	Intervention	2.0	2.5	+0.5	0.300
	control	2.0	2.4	+0.4	0.340
Hypertension	Intervention	3.5	4.5	+1.0	0.030
	control	3.0	3.2	+0.2	0.410
Women with Hypertension	Intervention	1.3	3.5	+2.2	0.015
	control	1.3	1.9	+0.6	0.220
Men with Hypertension	intervention	1.1	1.3	+0.2	0.450
	control	1.0	1.2	+0.2	0.470

**Medication adherence was assessed using the Medication Adherence Rating Scale (MARS; score range 0–10).*

†Δ indicates change between baseline and follow-up values.

MARS – Medication Adherence Rating Scale.

Table S 3. HbA1c by Medication Adherence

Adherence Group	N	Mean HbA1c (%)	SD
Adherent (MARS ≥6)	232	8.76	1.49
Non-adherent (MARS <6)	136	8.28	1.53

**Medication adherence was assessed using the Medication Adherence Rating Scale (MARS; score range 0–10). Participants with scores ≥6 were classified as adherent and <6 as non-adherent.*

†Values are presented as mean ± SD.

HbA1c – glycated hemoglobin; MARS – Medication Adherence Rating Scale; SD – standard deviation.

Table S 4. BP by Medication Adherence

Adherence Group	N	Mean BP (mmHg)	SD
Adherent (MARS ≥6)	174	152.46	10.39
Non-adherent (MARS <6)	349	151.94	10.75

**Medication adherence was assessed using the Medication Adherence Rating Scale (MARS; score range 0–10). Participants with scores ≥6 were classified as adherent and <6 as non-adherent.*

†Values are presented as mean ± SD.

BP – blood pressure; MARS – Medication Adherence Rating Scale; SD – standard deviation.

Table S5 in the Online Supplementary Document. Adjusted mean MARS scores and absolute differences between smartphone users and non-users at 6-month follow-up among participants with type 2 diabetes.

Group	Smartphone Users (Adjusted mean, 95% CI)	Non-Smartphone Users (Adjusted mean, 95% CI)	Absolute Difference (95% CI)
Type 2 Diabetes			
All with type 2 diabetes	0.996(0.923-1.069)	0.943(0.870-1.015)	0.053(-0.050, 0.156)
Men	0.946(0.841-1.051)	0.982(0.829-1.009)	-0.036(-0.188, 0.116)
Women	1.024(0.937-1.111)	0.919(0.829-1.009)	0.105(-0.030, 0.240)

Footnotes:

**Adjusted mean values were estimated using generalized estimating equations (GEE), accounting for clustering by site and repeated measurements within participants.*

†Models were adjusted for baseline MARS score, age, sex, and treatment facility.

Abbreviations:

CI – confidence interval, GEE – generalized estimating equations, MARS – Medication Adherence Rating Scale.

Table S6 in the Online Supplementary Document. Adjusted mean MARS scores and absolute differences between smartphone users and non-users at 6-month follow-up among participants with hypertension.

Group	Smartphone Users (Adjusted mean, 95% CI)	Non-Smartphone Users (Adjusted mean, 95% CI)	Absolute Difference (95% CI)
Hypertension			
All with hypertension	1.153(1.034-1.272)	1.078(0.953-1.202)	0.075(-0.097, 0.247)
Men	1.220(1.019-1.421)	1.234(1.031-1.437)	-0.014(-0.295, 0.267)
Women	1.102(0.954-1.250)	0.978(0.825-1.130)	0.124(-0.094, 0.342)

Footnotes:

**Adjusted mean values were estimated using generalized estimating equations (GEE), accounting for clustering by site and repeated measurements within participants.*

†Models were adjusted for baseline MARS score, age, sex, and treatment facility.

Abbreviations:

CI – confidence interval, GEE – generalized estimating equations, MARS – Medication

Appendix S1: Background information about the interactive mHealth application (AfyaPro connected care)

AfyaPro connected care is a comprehensive healthcare management platform, seamlessly integrating a spectrum of features. This all-encompassing solution includes patient engagement tools, clinical care plans, rule-based workflows, decision support, patient education, communication, logistics, finance, and data warehousing. Its strength lies in enriched content derived from best practices and expertise in behavioural change approaches, making AfyaPro a resilient solution for diverse healthcare needs.

A notable upgrade is the incorporation of telemedicine and remote-control features, representing a significant leap forward. This addition not only facilitates patient empowerment but also enables proactive remote care, solidifying AfyaPro's status as an integrated and forward-thinking healthcare program. With its tailored support for various aspects of healthcare delivery, AfyaPro emerges as a versatile and impactful tool, serving the needs of both healthcare professionals and patients effectively.

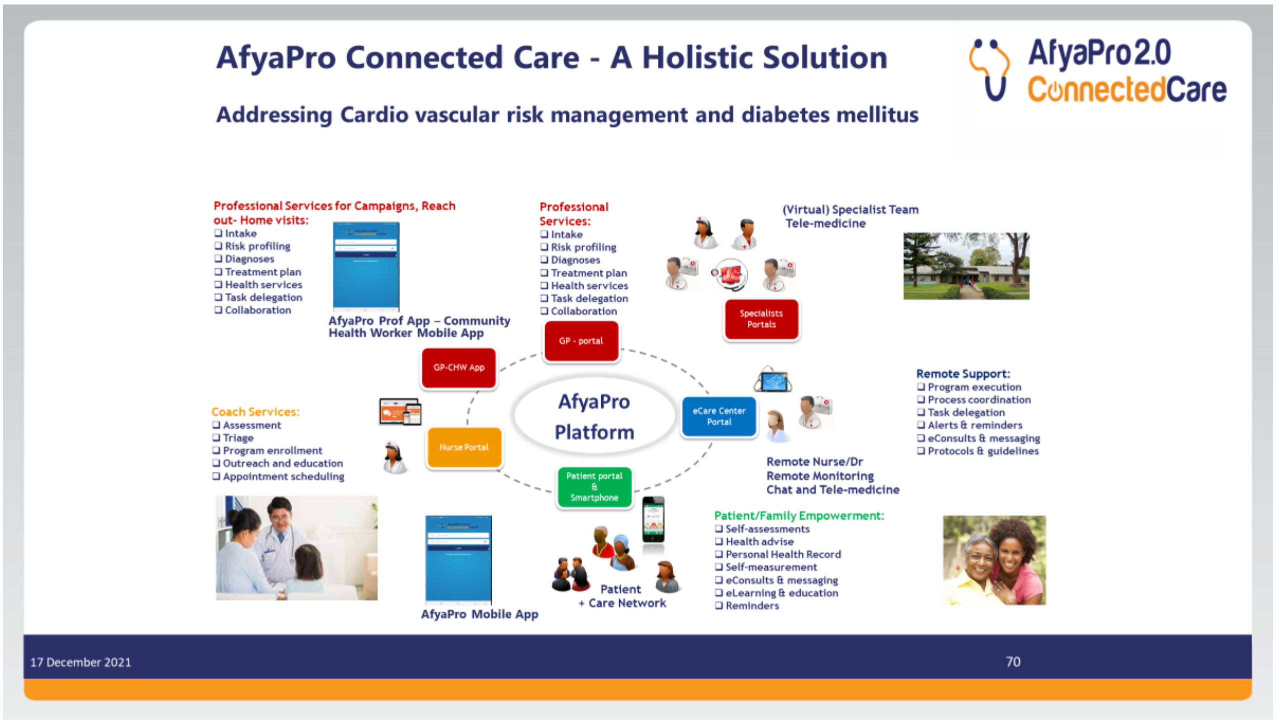

Figure 1; Afyapro Connected care Platform

Implementation phases of the interactive mHealth application

Step 1-Test the configured AfyaPro site and mobile App
Step 2-Assure all equipment is in place, networking done and connectivity is established
Step 3-The staff is trained on AfyaPro.
Two super users are identified and additionally trained.
Nearby IT support is defined and contracted to assist as needed.
Step 4-A plan for the migration of medical data is made
And put in motion. (Easiest is for the whole Clinic to migrate to AfyaPro)
Alternatively, experience can be obtained with AfyaPro for the CVRM-DM Clinic only.
A data clerk types over data as needed. As soon as the management of the Clinic is at ease with AfyaPro, the full migration can be done. Please note that integration is complex to maintain and expensive, could only be the case for a few selected EHRS).
Step 5-Final test of AfyaPro before going live

Step 6 -Enrol patients in AfyaPro. The enrolment of the patient in the CVRM-DM module is gradual. The nurse takes the vitals, patient history and does the risk scoring. Nurse indicates required lab test. Patient goes to lab for basic measurement Patient goes for doctor's visit.

(Some additional lab tests might be needed with a revisit to the doctor)

Step 7-After the visit, the doctor re- introduces the patient to the nurse practitioner for education and a new appointment. Nurse might assign care tasks. (Doctor can bring patient physically to nurse or send a communication through AfyaPro). The nurse enrols the patient in the follow-up program that contains a care plan – and includes the use of the mobile App (by the patient or by family members).

Optional: To standardize further, the notion persona can be very effective (see Phase 1, step 11). Based on questionnaire a 'persona' is assigned to a patient. A persona describes the possible behaviour of the patient, and the care plan/ treatment can be adjusted accordingly. Based on the persona and the risk profile, the patient is introduced by the nurse to a follow-up program. Standard will be a follow-up consultation with the doctor every three months (to be decided by the doctor).

Note: In parallel arrangements can be doctor with nutritionists to complement the activities of the nurse. Even coaching and buddy groups can be considered that are moderated by the nurse or a coach. This should be defined during phase 1, step 9 or 10.

Step 8- Working session between doctor and nurse practitioner. To review patients as needed. For doctor to verify the work of the nurse practitioner (medical data, care plan, care tasks). For nurse practitioner to have her questions clarified.
Step 9 – Continuous activity – Dr to patient relation (patient centered), An important and continuous activity is the doctor showing his (digital) presence to the patient. The doctor- patient bond needs to remain strong. (This could be through the chat function in the mobile app)
Step 10 – Continuous monitoring. Measure the results (population management module in AfyaPro) and make the evidence clear, including feedback to the patients and their family.

Step 11- Evaluate and refine step 6 to 10 of phase 2 and improve accordingly to prepare phase Hand over officially, define follow up needs. (The AfyaPro-IDBH support will apply in phase 3 but is bound to maximum levels.

Note: During this phase the AfyaPro-IDBH contracted research staff will visit once or twice and conducts interviews to learn on satisfaction, points of improvement and impact.

BACKGROUND READING SOME ILLUSTRATIONS OF AFYAPRO

Figure2 below, visually represents the versatile application of AfyaPro, showcasing its adaptability across various care programs and referral chain contexts. This illustration provides a clear understanding of how AfyaPro seamlessly integrates into different healthcare settings.

In Figure 3 below, a practical example demonstrates AfyaPro's role in supporting patient empowerment and remote care within an integrated proactive program. This visual representation offers insight into the platform's real-world implementation and its impact on patient care.

For a closer look at AfyaPro's user interface, Figure 4 below, presents a screenshot of the patient app. This glimpse into the interface provides a visual understanding of the

platform's design and functionality. Additional screenshots can be explored in Annex 2 for a more comprehensive view.

BACKGROUND READING SOME ILLUSTRATIONS OF AFYAPRO

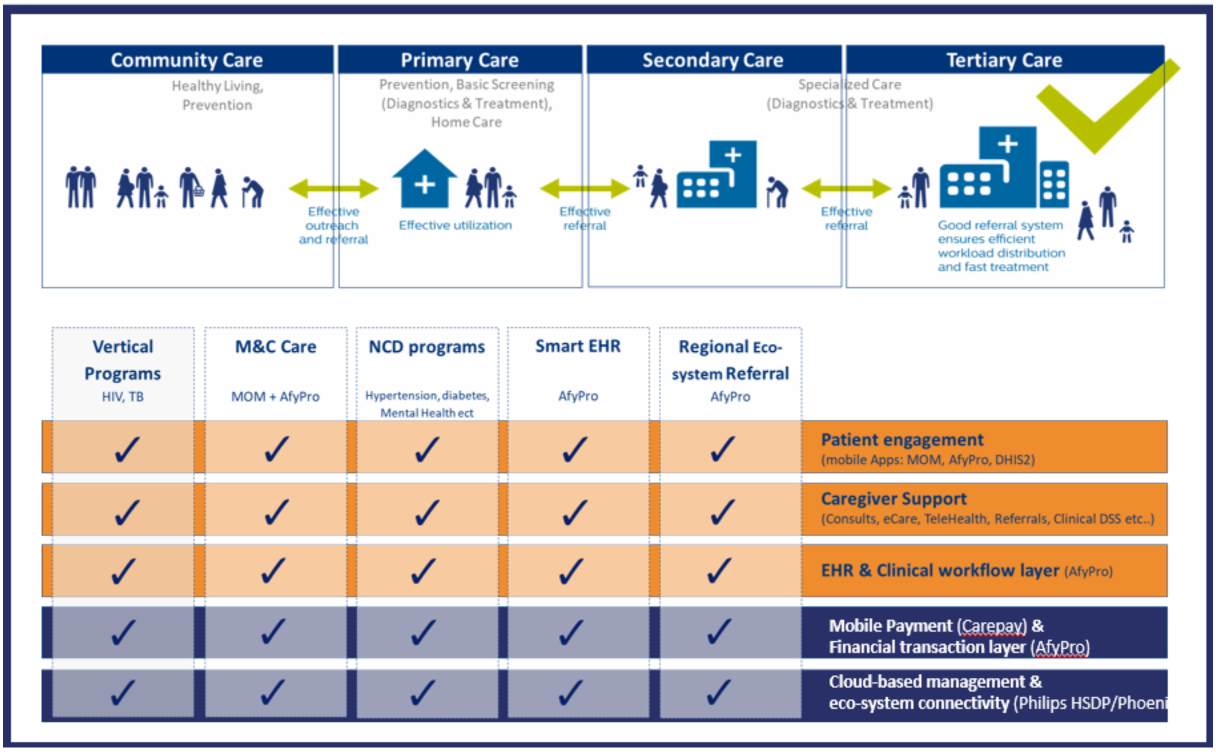

Figure 2: Applicability of AfyaPro for different care programs and referral chain contexts.

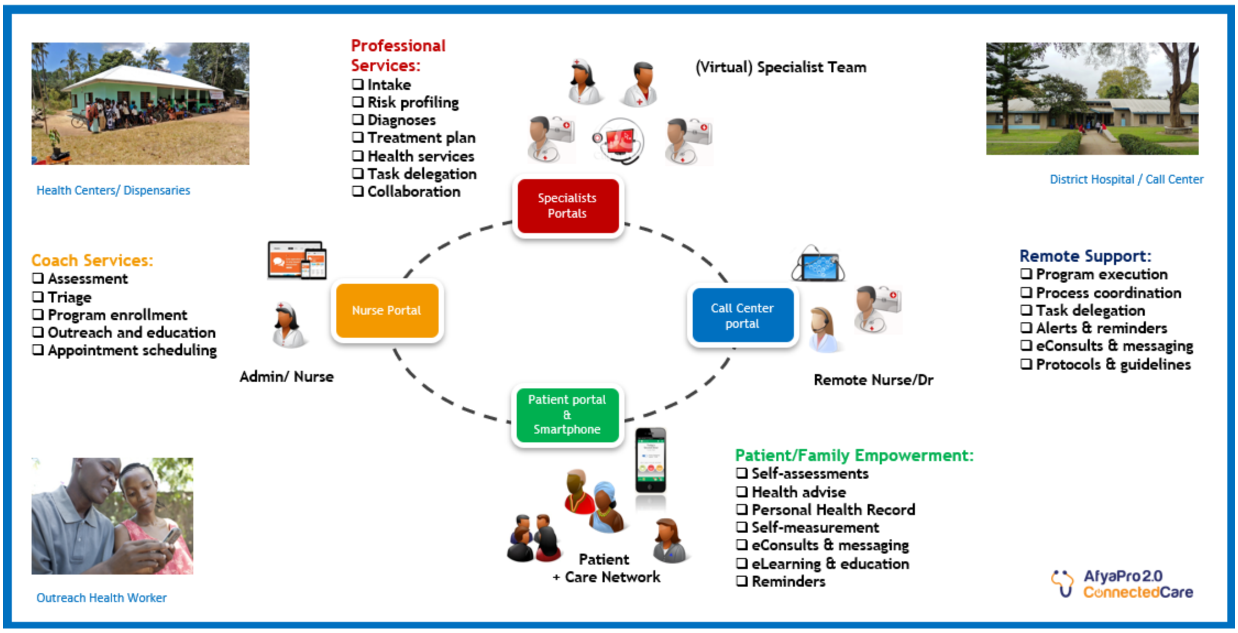

Figure 3: Example AfyaPro used to support patient empowerment and remote care from an integrated proactive program perspective.

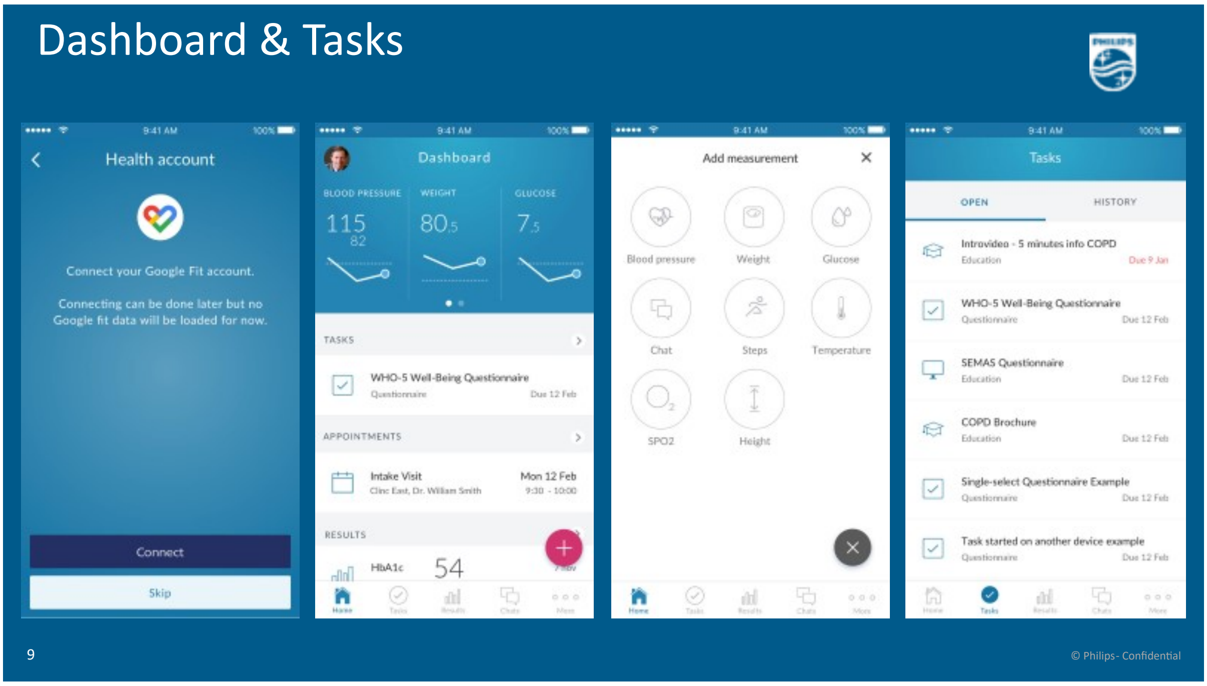

Figure 4: Screenshot of the patient App, for more screenshots see annex 2.

An important new feature for patients in this solution is telemedicine and remote control.

Appendix S2: Impact assessment of community empowerment and mobile health technology on diabetes and hypertension patient care management. (Baseline Adherence questionnaire)

Date of interview:	DD	MM	YYYY

Respondent ID:	Phone No:	Device ID *(intervention facilities)*:

Facility: [1] Kwahu Government Hospital [2] Weija -Gwabe Hospital [3] Sukura Hospital

Patient type: [1] Diabetes (Type II) [2] Hypertension

A. RESPONDENT'S BACKGROUND

1. Age (in years):	
2. Sex:	[1] Female [2] Male
3. Marital status:	[0] Never married [2] Married [4] Separated<br>[1] Cohabiting [3] Divorced [5] Widowed
4. Ever had biological children:	[0] No [1] Yes, how many surviving children?
5. Religion:	[0] None [1] Christian [2] Muslim [3] Traditional African<br>Other:
6. Ethnicity:	
7. Highest level of education completed:	[0] None [1] Primary [2] Secondary [3] Tertiary Other:
8. Employment:	[0] Not currently employed, **skip to Q12**<br>[1] Retired, **skip to Q12**<br>[2] Currently employed
9. Occupation:	[1] Clerical/Secretarial [4] Skilled manual craftsmanship<br>[2] Professional/Technical/Managerial [5] Unskilled manual labour<br>[3] Sales and services [6] Agricultural<br>Other:

10. Does your occupation earn you income?			[0] No		[1] Yes	
11. If yes, how often do you get income from your occupation?<br>[1] Daily [2] Weekly [3] Monthly [4] Quarterly [5] Yearly [6] Irregularly						
12. Do you depend on anyone for your financial upkeep?				[0] No		[1] Yes
13. If yes, who do you depend on? *(Multiple allowed)*<br>[1] Partner [1] Parent(s) [1] Child(ren) [1] Other family<br>[1] Friends<br>Other:						
14. Residence: [1] Within facility community [2] Outside facility community						
15. Length of stay at current residence: year(s), month(s), week(s), day(s)						
16. How long does it usually take you to get to this facility?<br>[1] Less than 30 minutes [2] 30-60 minutes [3] More than 1 hour						
17. Do you have health insurance? [0] No [1] Yes<br>If yes,<br>a. What type of health insurance? [1] NHIS [2] Private<br>b. Are you an active paid-up member? [0] No [1] Yes, confirmed [2] Yes, unconfirmed						

B. MOBILE DEVICE USE AND EXPERIENCES

18. Have you ever used a smart phone or tablet? [0] No [1] Yes<br>19. Do you own a mobile device? [0] No [1] Yes<br>If yes,<br>a. Which of the following mobile device(s) do you own? *(Multiple allowed)*<br>[1] Simple phone [1] Smart phone [1] Tablet<br>b. How long have you used the oldest of these device(s) for? _____ year(s), _____ month(s)<br>c. Do you have internet access through your mobile phone/device? [0] No [1] Yes

d. Which of the following do you usually use your phone for? *(Multiple allowed)*

[1] Phone calls (voice)   [1] Social Networking (WhatsApp, Facebook, etc.)
[1] SMS/MMS   [1] Entertainment (movies, videos, music, etc.)

Other:

20. Have you ever used a mobile device to access healthcare services?   [0] No   [1] Yes

If yes,

a. Was it your own device?   [0] No   [1] Yes

b. What did you use the device for? *(Multiple allowed)*

[1] Call/Text ambulance   [1] Call/Text healthcare worker   [1] Access e/mHealth services

Other:

21. If you could input your vitals and blood sugar levels into a mobile device for monitoring by this facility	No	Yes
a. Do you think you would input your information on a regular basis?	0	1
b. Would this make you feel more secure about your treatment?	0	1
c. Would you want the facility to contact you if there are problems seen with your data inputs?	0	1
d. Would you want to contact the healthcare workers yourself to ask any questions about how to best manage your condition through this medium?	0	1
e. Do you think this would help you in managing your disease condition?	0	1
f. Do you feel it may make you too reliant on the healthcare workers, i.e. make you less independent?	0	1

g. How much interaction would you want with the healthcare workers?

[1] Never   [2] Occasionally   [3] Sometimes   [4] Often   [5] Always

h. If this meant you would see a doctor less regularly, would this be a positive or a negative thing for you? [1] Negative [1] Positive
i. How willing would you be to pay for the ability to store all your information electronically, and have a health worker help you monitor your condition every month?<br>[1] Not at all [2] A little [3] Moderately [4] Mostly [5] Completely willing

C.ADHERENCE *(Based on Medication Adherence Rating Scale, MARS)*

	No	Yes
22. Do you ever forget to take your medication?	0	1
23. Are you careless at times about taking your medication?	0	1
24. When you feel better, do you sometimes stop taking your medication?	0	1
25. Sometimes if you feel worse when you take the medication, do you stop taking it?	0	1
26. I take my medication only when I am sick	0	1
27. It is unnatural for my mind and body to be controlled by medication	0	1
28. My thoughts are clearer on medication	0	1
29. By staying on medication, I can prevent getting sick.	0	1
30. I feel weird, like a 'zombie' on medication	0	1
31. Medication makes me feel tired and sluggish	0	1

32. Where do you usually get medications for your condition from?
    [1] In this facility [2] In a different facility, state type:_________________________

C. ANTHROPOMETRICS AND BIOMARKERS

To be repeated monthly, quarterly or biannually.

Date of interview:	DD	MM	YYYY

Respondent ID:	Phone No:	Device ID *(intervention facilities)*:

33. Anthropometric Measurements and Biomarkers

Height (cm)			HBA1C first reading	
Weight (kg)			HBA1C second reading	
Waist circumference (cm)			Total cholesterol (mmol/L)	
Hip circumference (cm)			LDL (mmol/L)	
Systolic BP (mmHg)	1st	2nd	HDL (mmol/L)	
Diastolic BP (mmHg)	1st	2nd	Triglycerides (mmol/L)	

Appendix S3 in the Online Supplementary Document. Smartphone usage logs for November 2022.

Smartphone_and_Health_App_Usage_Data

Participant	Date	Hours Used	Activity Type	Feature Used	Frequency of Use	Total Usage (hrs)
P001	11/01	1.5	Health App Usage	Monitoring Reminder	Daily	3.5
P001	11/05	2.0	Health App Usage	Clinic Appointment	Weekly	5.0
P001	11/08	2.0	General Smartphone Usage	Monitoring Reminder	Twice Weekly	5.5
P001	11/12	2.0	Health App Usage	Monitoring Reminder	Daily	7.5
P001	11/18	2.5	Health App Usage	Clinic Appointment	Weekly	10.0
P002	11/05	1.5	General Smartphone Usage	Monitoring Reminder	Every Other Day	2.5
P002	11/08	2.5	Health App Usage	Clinic Appointment	Weekly	5.0
P002	11/15	2.5	Health App Usage	Monitoring Reminder	Daily	7.5
P002	11/22	2.5	General Smartphone Usage	Clinic Appointment	Weekly	10.0
P002	11/30	1.0	Health App Usage	Monitoring Reminder	Daily	2.0
P003	11/02	2.0	General Smartphone Usage	Clinic Appointment	Weekly	4.0
P003	11/07	2.0	Health App Usage	Monitoring Reminder	Every Other Day	6.5
P003	11/14	2.5	Health App Usage	Monitoring Reminder	Twice Weekly	9.0
P003	11/20	2.5	General Smartphone Usage	Clinic Appointment	Weekly	9.0
P003	11/28	2.0	Health App Usage	Monitoring Reminder	Daily	11.0
